# The evolution of biocontainment unit care for high-consequence infectious diseases: progress, lessons learned, and future directions

**DOI:** 10.1186/s13054-026-06259-4

**Published:** 2026-08-15

**Authors:** Elizabeth Chasen, Indrani Biswas, Anthony Lo Piccolo, Ryan Leone, Erin McGuire, Vikramjit Mukherjee, Radu Postelnicu

**Affiliations:** 1 NYC Health + Hospitals/Bellevue, New York, NY USA; 2 UPMC Pittsburgh, Pittsburgh, PA USA; 3 NYU Grossman School of Medicine, New York, NY USA; 4 Vagelos College of Physicians and Surgeons, Columbia University, New York, NY USA

**Keywords:** Biocontainment, Biocontainment unit, High consequence infectious disease, Critical care, Ebola virus

## Abstract

Biocontainment units (BCUs) are specialized facilities designed to treat patients with high-consequence infectious diseases (HCIDs) which are pathogens with high mortality rates, limited treatment options, and significant potential for healthcare-associated transmission. Since the opening of the first modern BCU in 1971, these units have evolved from simple isolation environments into advanced centers capable of delivering comprehensive critical care while maintaining rigorous infection control. This evolution has been driven from the lessons learned from past outbreaks of Ebola, Marburg, Middle Eastern Respiratory Syndrome (MERS), Severe Acute Respiratory Syndrome Coronavirus 2 (SARS-CoV-2), among other emerging infectious threats. Four key domains contribute to the care of patients with HCIDs – laboratory capabilities, critical care delivery, healthcare workforce readiness, and safe patient transport. Improvements in diagnostic capabilities have allowed for earlier disease recognition while greater access to routine laboratory tests better inform clinical decision making. Clinical care capabilities now match those seen in intensive care units while maintaining infection control standards. Training of specialized workforce and multidisciplinary staffing models have improved bedside care and operational readiness. Meanwhile, innovations in ground and air transport have enhanced the ability to safely transfer HCID patients to facilities best suited to provide care. This review synthesizes the evolution of BCU capabilities globally, examining advances in critical care, diagnostics, investigational therapeutics, workforce models, and patient transport.

**Graphical abstract:**

**Figure:**
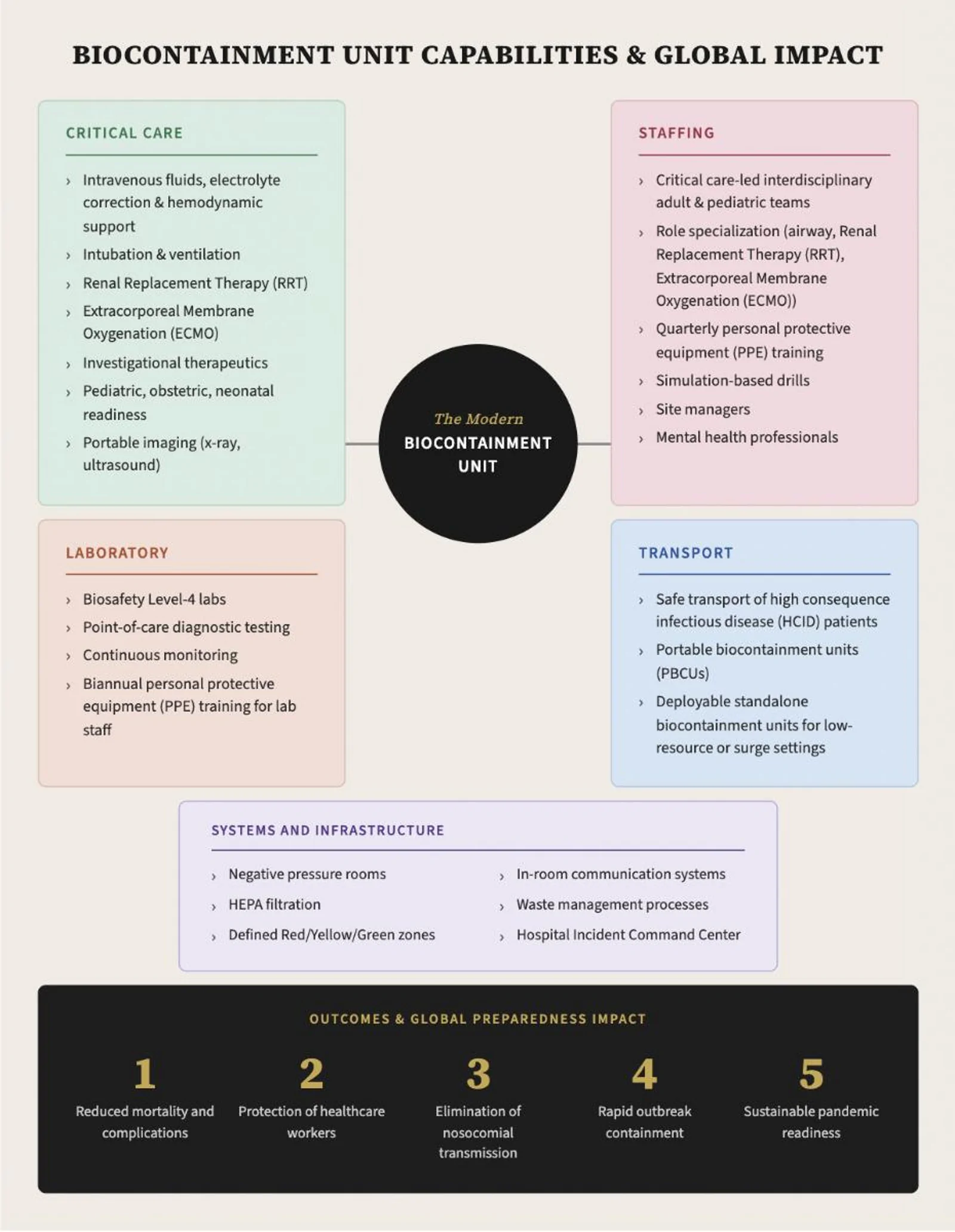

## Background

### Origins of BCUs

Biocontainment units (BCUs) are specialized facilities dedicated to treating patients with high-consequence infectious diseases (HCIDs), including viral hemorrhagic fevers (VHFs) like Ebola virus disease (EVD). BCUs sit at the intersection of biosafety science, critical care medicine, and public health preparedness. The first modern BCU opened in 1971 in the U.S. Army’s Medical Research Institute of Infectious Diseases (USAMRIID) as a two-bed facility designed to manage accidental exposures to highly hazardous pathogens. During its 41-year existence, this unit housed a total of 21 patients exposed to highly hazardous infectious diseases, such as Lassa Fever and EVD, though none ultimately became infected [1]. Subsequently, in the early 2000s, BCUs opened at civilian academic medical centers, including at Emory University, the University of Nebraska, and New York City Health + Hospitals/Bellevue (NYC H + H/Bellevue) [1]. These three BCUs became the foundation of the United States (U.S.) HCID preparedness and response after treating patients during the 2014–2015 EVD epidemic. Today, they co-lead the National Emerging Special Pathogen Treatment and Education Center (NETEC) and are among the 13 federally-designated Regional Emerging Special Pathogen Treatment Centers (RESPTCs). RESPTCs operate BCUs and serve as national leaders in HCID preparedness and response, significantly expanding national capacity for HCID management [2]. Since 2015, U.S. RESPTCs have also been activated for early COVID-19 patients, individuals with Mpox, and patients suspected of an HCID infection [3, 4]. 

BCUs have expanded from isolation-focused environments to units capable of delivering a full spectrum of organ support tailored to HCID physiology, including on-unit Bio-safety Level 3 (BSL-3) clinical laboratories that provide 24/7 monitoring of patients’ body fluid markers and organ function [5]. Advances in biosafety science, international outbreaks, and changes in public health policy also shaped the evolution of biocontainment units. Engineered controls, such as negative pressure rooms, anterooms, directional airflow, high-efficiency particulate air (HEPA)-filtered ventilation systems, and airtight construction, are designed to ensure that pathogens are contained while enabling high-acuity care [5]. Space planning includes “hot” or contaminated zones, dedicated donning and doffing corridors, and delineated clean zones to enforce proper usage of personal protective equipment (PPE) [6]. Controlled movement of staff, patient, and waste through restricted routes minimizes cross-contamination.

The 2001 U.S. anthrax attacks further accelerated federal investment in hospital preparedness in the U.S. Parallel international efforts led to the establishment of BCUs across Europe, Canada, and Australia. Anthrax cannot be transmitted human-to-human; however, a proper isolation space during the patient evaluation phase (if anthrax exposure is unknown), advanced critical care, and BSL-3 laboratory testing are needed for these patients, making BCUs desirable for management of anthrax patient. Together, these initiatives integrated BCUs into broader national and global biodefense strategies linking clinical care, surveillance, emergency response, and research [7–9]. 

BCU infrastructure has advanced across the world including in Asia and Europe. Since 1975, Princess Margaret Hospital (PMH) has served as Hong Kong’s designated infectious disease center; however, when the 2003 SARS epidemic hit, the hospital had few isolation spaces existed, no ante rooms, and insufficient ventilation [10]. This was a catalyst for the Hong Kong government to invest substantial funding in emerging infectious disease infrastructure including over 1,400 isolation beds, and negative pressure rooms [11]. Today, PMH’s Infectious Disease Centre includes 14 intensive care beds, eight floors of negative-pressure wards, segregated clean and contaminated circulation, HEPA-filtered ventilation, and a Biosafety Level 3 laboratory [12]. The Japan Institute for Health Security (JIHS) was established in 2025 to strengthen Japan’s capacity to respond to HCID threats. It is a merger between the National Center for Global Health and Medicine (NCGM), which has 749 beds including a four bed BCU, and the National Institute of Infectious Diseases (NIID), which is the federal agency responsible for infectious disease response, research and therapeutic developments [13]. And in South Korea, the Korea Centers for Disease Control and Prevention (KCDC) was established in 2004 and invested in nation-wide BCU infrastructure, and manages antiviral stockpiles [14]. 

Collaboration amongst Germany’s BCU’s began as early as 1999 in response to yellow fever, and formalized in 2014 in response to the Ebola epidemic. The German Permanent Working Group of Competence and Treatment Centers for High Consequence Infectious Diseases (STAKOB) is composed of 8 public health authorities and 7 treatment centers ready to respond to HCID cases [15]. England has had a longer history of BCU care with its first case of Ebola Virus disease in 1976. They utilized a Trexler flexible-film isolator which is a negative pressure, isolation tent that allows staff to provide care through a half suit imbedded into the side wall. This system is still utilized today with benefits including minimizing HCID PPE use (reducing exposure risk associated with donning and doffing), and easier training allowing for a larger multidisciplinary team. Today, England’s HCID clinical network includes 4 BCUs designated for pathogens transmitted by direct contact with bodily fluids and 7 BCUs designated for pathogens transmitted by airborne routes [16]. While many first world countries have enhanced their BCU capabilities, low-to-middle income countries still struggle to reach similar abilities.

Early BCU development also necessitated the application of established ethical principles to novel challenges arising from the unique context of high-consequence infectious disease care. Ethics committees addressed challenges such as balancing strict infection prevention measures—including restricting family visitation—with patients’ psychosocial needs of patients and families. They also reckoned with questions about whether healthcare workers could be permitted to opt out of caring for patients with these diseases. The development of BCUs also required new approaches to workforce protections and operational governance that differed from conventional hospital practice. The need to balance rigorous containment with humane, patient-centered care became a defining principle in the maturation of BCUs and influenced subsequent national and international guidelines for high-level isolation care [17, 18]. 

**Fig. 1:**
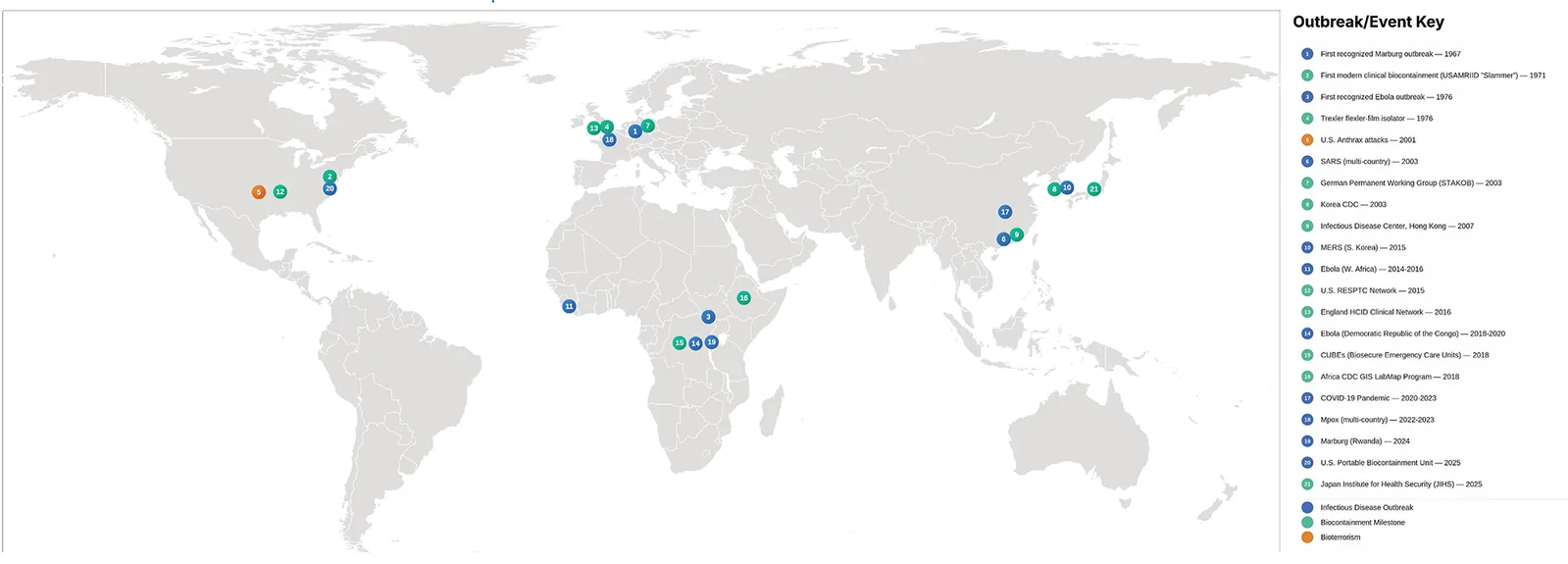
Key Outbreaks and Advancements in HCID Care. Geographic distribution of key outbreaks and events shaping BCU development, numbered chronologically as in Table 1. DRC= Democratic Republic of Congo

In this review, the authors first selected key domains contributing to BCU care: critical care capabilities, investigational therapeutics, and healthcare worker team formation and training models, and patient transport. Then a literature review was conducted in PubMed to identify key milestones advancing practice within each domain. The key milestones that have driven BCU development from the first recognized viral hemorrhagic fever outbreaks through to the present day are summarized below in Table 1, and their geographic distribution is shown in Fig. 1. This manuscript describes the evolution of BCU care and analyzes existing care limitations alongside practical substitutes.

**Table 1:** Key Outbreaks and Events Shaping the Evolution of Biocontainment Units

ID	Year(s)	Outbreak/Events	BCU Advancement	Why it mattered
1	1967	First recognized Marburg outbreak reported in Germany	Early “high-risk pathogen” clinical isolation thinking; containment care models	Established the need for specialized isolation for VHFs
2	1971	First modern clinical biocontainment (USAMRIID “Slammer”)	Proof-of-concept for high-level clinical containment; engineered isolation + controlled workflows; “zero secondary infections” despite exposures	Demonstrated containment can coexist with clinical care
3	1976	First recognized Ebola outbreak reported in South Sudan and the Democratic Republic of the Congo (DRC)	Learnings from poor IPC practices. Confirmed patients isolated	Exposed poor local hospital IPC practices which exacerbated the outbreak, while demonstrating effectiveness of contact tracing and isolation in reducing transmission
4	1976	England’s Trexler flexible-film isolator	A novel isolation unit that includes negative pressure, HEPA filtration, and a “half suit” for healthcare staff	Minimized exposure risk, less intense training allows for a large multidisciplinary team
5	2001	U.S. Anthrax attacks	Accelerated hospital preparedness funding and biodefense infrastructure; formalized surge planning + IPC investments	Policy-driven expansion of hospital-based biodefense capacity
6	2003	SARS (multi-country)	Modernized hospital isolation readiness: stricter IPC workflows, airborne-precaution thinking, training discipline	Forced hospitals to operationalize high-level isolation at scale
7	2003	German Permanent Working Group of Competence and Treatment Centers for High Consequence Infectious Diseases (STAKOB)	The establishment of a network of 8 public health authorities and 7 treatment centers ready to respond to HCID cases	A model for integrating geographically dispersed public health authorities and hospitals within a coordinated national network
8	2004	Korea Centers for Disease Control and Prevention (KCDC)	South Korea invested in nation-wide BCU infrastructure	Laid the groundwork for South Korea to later become a globally recognized leader in COVID-19 response
9	2007	Infectious Disease Center, Princess Margaret Hospital (Hong Kong)	Establishment of an infectious disease center with major infrastructure improvements in response to 2003 SARS outbreak	A model for expanding surge capacity within an existing frontline hospital
10	2015	MERS (South Korea)	Reinforced governance around hospital transmission, controlled movement, and cohorting/isolation capacity	Highlighted how hospital systems amplify outbreaks if IPC fails
11	2014–2016	West Africa Ebola	Shift from “futility” to evidence-based supportive care; PPE + safety systems; catalyzed U.S. BCU network strengthening (NETEC era)	Cemented BCUs as critical-care-capable containment units
12	2015	U.S. RESPTC Network	The establishment of 10 biocontainment units across the United States	Increased the U.S. BCU bed capacity and allowed for the formation of dedicated, multidisciplinary teams to advance BCU care capabilities
13	2016–2018	UK/England HCID Clinical Network	Establishment of separate HCID treatment centers for airborne HCIDs and contact HCIDs	Formalized a national model that differentiates HCID treatment capacity according to transmission route
14	2018–2020	DRC Ebola (North Kivu/Ituri)	Improved integration of supportive care + clinical trials infrastructure; more mature outbreak clinical systems	Showed outcomes improve when supportive care and systems mature
15	2018	CUBEs (Biosecure Emergency Care Units for Outbreaks) (DRC)	Alliance for International Medical Action (ALIMA) set up innovative mobile BCUs called CUBEs (Biosecure Emergency Care Units for Outbreaks)	Significantly reduced mortality among Ebola patients in the DRC, while improving healthcare worker safety and efficiency, and the patient experience of isolation
16	2018-present	Africa CDC GIS Laboratory Mapping (LabMap) Program	A continent-wide system for geospatially mapping laboratory capacities, capabilities, and networks to identify gaps and inform planning for diagnostic and outbreak-response capacity	Created a data-driven approach to strengthening Africa’s laboratory network by allowing countries to visualize geographic gaps in diagnostic capacity, optimize resource allocation, and develop interconnected laboratory systems across borders
17	2020–2023	COVID-19 Pandemic	Surge staffing models, tiered care, workflow redesign; scaling of advanced support (ventilation/ECMO in experienced centers); systems coordination	Proved outbreak care depends on staffing systems as much as beds
18	2022–2023	Multi-country Mpox outbreak in non-endemic countries	Reinforced HCID pathways beyond filoviruses; flexible isolation models and IPC operations for non-VHF threats	Expanded the “BCU mindset” to broader special pathogens
19	2024	Rwanda Marburg outbreak	Demonstrated improvement of advanced ICU-level care in West Africa; reinforces “safe high-acuity care without nosocomial spread” theme	Powerful modern example that advanced care is feasible in outbreak settings
20	May 2025	U.S. Portable Biocontainment Unit (PBCU) milestone	Adds “mobile containment + continuous care during transport” to the BCU ecosystem	Connects outbreak care to transport logistics and national systems
21	2025	Japan Institute for Health Security (JIHS)	Established through merger of the National Center for Global Health and Medicine and the National Institute of Infectious Diseases, JIHS integrates infectious disease intelligence, research and development, clinical services, and workforce development under one national institution	Distinctive model for integrating scientific advisory capacity, disease intelligence, basic and clinical research, and comprehensive medical response within a single institution to provide coordinated guidance during HCID outbreaks

IPC = Infection Prevention and Control; VHF = Viral Hemorrhagic Fever; HCID = High-Consequence Infectious Disease; DRC= Democratic Republic of the Congo; NETEC = National Emerging Special Pathogen Treatment and Education Center; ECMO = Extracorporeal Membrane Oxygenation; PBCU = Portable Biocontainment Unit

### Insights from EVD and Marburg: the central role of supportive and critical care

During the 2014–2016 EVD epidemic in West Africa, no pathogen-specific therapies had demonstrated efficacy, and clinical trials of investigational agents began only late in the epidemic [19]. Consequently, supportive care represented the primary therapeutic intervention. Higher-resource hospitals in the U.S. and Europe were able to provide more intense supportive and critical care than hospitals in West Africa due to differences in caseload and available resources. All patients in the U.S. or Europe received oral or intravenous electrolyte replacement to correct metabolic abnormalities with a majority of patients receiving antiemetics, empirical antibiotics, and supplemental oxygen. Mortality among these patients were 18.5% compared to 37–74% in West African treatment units during this outbreak [20]. Confounding this finding, 85% of patients in Europe and the U.S. received investigational therapeutics, however the clinical benefit could not be assessed or separated from the impact of supportive care. The limited capability to provide robust supportive care in West Africa possibly led to preventable deaths. Delivering these interventions in the context of a highly contagious and lethal pathogen posed unprecedented challenges, including uncertainty surrounding transmission risk, limited knowledge of infectious dose, and concerns regarding healthcare worker safety during high-risk procedures.

In 2018, the rural North Kivu region of the Democratic Republic of the Congo (DRC) experienced a large EVD epidemic and benefited from improved supportive and critical care measures, such as vasopressors and non-invasive positive pressure ventilation. In 2018, the nongovernmental organization (NGO) Alliance for International Medical Action (ALIMA) set up innovative mobile BCUs called CUBEs (Biosecure Emergency Care Units for Outbreaks) in the DRC that are certified at BSL-4 and equipped with climate control and continuous observation capabilities. These interventions contributed to a lower mortality rate (~ 40%) compared to the first outbreak (~ 80%) [21, 22]. 

Better supportive care in higher-resource settings was also associated with better outcomes in Marburg Virus Disease (MVD). While MVD outbreaks in rural areas had fatality rates of 80–90% [23], the 2024 Rwanda MVD outbreak had a significantly lower fatality rate of 23%, in large part due to its emergence in two large hospitals in the capital city, Kigali, where care coordination, advanced interventions, and investigational therapies were available [23, 24]. 

Collectively, these observations from EVD and MVD outbreaks demonstrate that outcomes are not solely dictated by pathogen virulence, but also by the capacity to deliver timely, high-quality supportive and critical care. The consistent association between improved survival and access to intravenous resuscitation, electrolyte monitoring, organ support, and multidisciplinary critical care underscores the central role of healthcare system preparedness—even outside of high-income countries.

## Main text

### Modern diagnostic and laboratory capabilities

Reliable, real-time diagnostics are foundational to BCU care, enabling clinical decision-making without compromising infection control. A defining feature of modern BCUs is the integration of point-of-care diagnostic testing, which allows frequent monitoring of laboratory values without the need for centrifugation, thereby reducing aerosolization risk [25]. In 2025, 11 out of 13 RESPTCs (84%) reported having a dedicated laboratory within their BCU [5], and a survey of 22 BCUs in the U.S. and Europe found that laboratory testing is performed within the BCU in 63% of centers, within the patient room in 21%, and outside the unit in just 16% [25]. Beyond laboratory testing, modern BCUs are equipped with portable imaging technologies that enable bedside assessment while clinicians remain in full PPE. Mobile radiography systems, dedicated ultrasound platforms, and digital stethoscopes with disposable earpieces facilitate comprehensive cardiopulmonary evaluation [6]. The EVD outbreak accelerated the adoption of point-of-care ultrasound (POCUS) as a standard-of-care tool for vascular access and procedural guidance, substantially reducing procedure time, limiting repeated needle sticks, and decreasing provider exposure [26]. 

Significant progress has been made in expanding diagnostic capacity globally. Since 2015, African countries have deployed rapid diagnostic tests for human immunodeficiency virus (HIV), tuberculosis, malaria, and COVID-19, and portable molecular technologies now enable on-site detection and sequencing even in remote areas, reducing delays in diagnosis and improving outbreak responses. Despite these gains, persistent challenges remain, including heavy reliance on external funders, imported equipment, and coordination gaps among partners [27]. Initiatives such as the Africa Centres for Disease Control and Prevention (CDC) Laboratory Systems and Network Capacities Framework aim to address these gaps by expanding geospatial mapping of laboratory networks and using this data to optimize resource allocation, strengthen surveillance, and inform evidence-based policies across national and regional systems [28]. Looking ahead within BCUs themselves, future directions include integration of artificial intelligence-assisted imaging interpretation and the development of compact, easily decontaminated imaging platforms. These innovations may shift reliance away from centralized imaging infrastructure toward decentralized, clinician-operated tools, improving both diagnostic speed and accessibility.

### The evolution of critical care delivery

The evolution of critical care delivery within BCUs reflects a progressive shift from cautious, resource-limited practice toward evidence-based, high-acuity management. Intravenous fluid resuscitation and vasoactive support represent the most fundamental interventions. During the 2014–2016 Ebola epidemic, these were severely limited in West Africa due to inconsistent availability of supplies and trained personnel. These experiences underscored the importance of central venous access and advanced hemodynamic management, particularly as peripheral intravenous lines frequently infiltrate in the context of severe dehydration [29]. Adjunctive strategies, including conscious sedation to limit patient movement and preemptive placement of invasive lines, further reduced emergency interventions and associated infection risks [30]. 

Renal replacement therapy (RRT) illustrates how BCU capabilities have expanded over time. In 2018, an international expert panel did not specify protocols for RRT in EVD patients, viewing it as beyond the scope of broadly applicable recommendations [31]. Yet despite this, 19% of Ebola patients in the U.S. and Europe received RRT for acute renal failure [20], and surveys conducted in both 2020 and 2023 found that 71% of U.S. Special Pathogen Treatment Centers were able to provide RRT to confirmed VHF patients in their BCUs [32, 33]. In general conventional intermittent hemodialysis would be preferred; however, continuous renal replacement therapy (CRRT) is most commonly used in BCUs since it requires less intensive staffing requirements and machine utilization. Also, CRRT would be preferred for significantly hypovolemic patients as could be expected from VHF patients. During the 2024 Marburg outbreak in Rwanda, RRT was available and highlighted as a potentially important component of care [34]. From a practical standpoint, RRT is preferable to conventional dialysis in resource-limited settings, as it can be delivered by continuous, self-contained portable units, requires fewer connections and disconnections, and minimizes staff exposure [35]. 

Airway management and mechanical ventilation have been among the most ethically and operationally contentious aspects of HCID care. Early in the EVD epidemic, clinicians across both high- and low-resource settings questioned whether noninvasive or invasive ventilation could be delivered safely to HCID patients [36, 37]. In response, clinicians initially restricted the use of bilevel positive airway pressure (BiPAP), high-flow nasal cannula, and endotracheal intubation. Even after it became clear that airborne transmission was not a significant route of Ebola virus spread, NETEC guidelines as recently as 2019 still did not recommend BiPAP or high-flow nasal cannula due to concerns about aerosol generation [38]. These restrictions were shaped in part by resource availability: in high-resource settings, 33% of Ebola patients ultimately received noninvasive or invasive mechanical ventilation, whereas such interventions were available in only one treatment unit in Sierra Leone and only late in the West African outbreak [21]. 

Over time, NETEC and other expert groups developed procedural guidance to minimize aerosolization risk during airway management [36]. Recommended strategies included semi-elective timing of intubation, rapid sequence induction with video laryngoscopy, strict avoidance of bag-valve-mask ventilation, HEPA filtration, prevention of circuit disconnections, and airway training while wearing full PPE. These approaches reframed intubation not as a prohibited intervention but as one requiring meticulous planning and specialized expertise. The same debates re-emerged during the early COVID-19 pandemic, when fear of aerosol transmission initially drove recommendations for early intubation and limited use of noninvasive support [28, 32]. Improved understanding of transmission dynamics ultimately led to the safe and widespread adoption of both invasive and noninvasive ventilation across pandemic settings [39].

Extracorporeal membrane oxygenation (ECMO) represents the leading edge of critical care capability within BCUs, and its trajectory mirrors the broader pattern of initial restriction followed by evidence-based expansion. During the EVD outbreak, ECMO was not utilized, reflecting concerns about coagulopathy, bleeding risk, and prolonged high-intensity bedside exposure. In 2014, the Extracorporeal Life Support Organization (ELSO) Steering Committee explicitly advised against its use in EVD patients, citing the absence of supporting evidence and the high risk to healthcare workers [40]. In contrast, ECMO became a cornerstone salvage therapy during the COVID-19 pandemic for patients with refractory acute respiratory distress syndrome, supported by rapidly evolving evidence and unprecedented system-level investment [41, 42]. Hospitals expanded ECMO capacity by standardizing platforms, stockpiling circuits, redeploying machines, training nontraditional staff, and developing centralized staffing and tracking systems [42], demonstrating that even the most resource-intensive critical care interventions can be safely integrated into outbreak response when supported by infrastructure, training, and coordinated systems. While there is no evidence to fully support ECMO as a primary modality to support patients with a VHF, its utilization may be vital for VHF patients with secondary insults such as bacterial pneumonia or cardiogenic shock. There is also evidence supporting the use of veno-arterial ECMO (VA-ECMO) for severe dengue where the insult is severe but time-limited [43]. Nevertheless, a 2023 survey found that only 28.6% of U.S. Special Pathogen Treatment Centers would perform ECMO on a VHF patient in their BCUs, indicating that significant institutional variation in willingness and readiness persists [32]. 

The medical counter measure (MCM) landscape for HCIDs remains limited. The rarity of HCID outbreaks, coupled with limited research infrastructure and funding in outbreak settings, has resulted in relatedly little research on HCID MCMs. However, in the last few years progress has been made to stand up research trails in outbreak settings. The Partnership for Research on Ebola Vaccines (PREVAC) trail was designed after the 2014–2016 EVD epidemic by a consortium of West African countries to assess the safety and efficacy, and durability of antibody responses of Ebola vaccine candidates [44]. Thanks to this trial, the first and only Ebola vaccine was approved by the U.S. Food & Drug Administration (FDA), and the European Medicines Agency’s Committee for Medicinal Products for Human Use (CHMP), with a global stock pile of over 500,000 doses managed by the United Nations International Children’s Emergency Fund’s (UNICEF’s) International Coordinating Group on Vaccine Provision [45, 46]. Additionally, monoclonal antibody cocktails have shown promising efficacy against Ebola Virus Sudan Strain, Ebola Virus Bundibugyo, and Marburg Virus; however, these therapeutics are still under investigation. As of July 2nd, 2026, monoclonal antibody MBP-134 is being evaluated for effectiveness in patients with Ebola Virus Bundibugyo in the DRC through the Platform Adaptive Randomized Trial for New and Repurposed Filovirus Treatments (PARTNERS) trial, sponsored by the World Health Organization (WHO) [47]. Continuing research on HCID MCMs is critically important so that safe, proven therapeutics are available for patients as soon as a new outbreak is identified. In the future, artificial intelligence may help expedite drug discovery by identifying potential targets quicker, and vaccine development by better predicting antigen protein structures [48]. 

Investigational therapeutics and vaccines have advanced in parallel with improvements in supportive care, though the relationship between the two is important to understand. Experimental therapies are best evaluated against a backdrop of consistently high-quality supportive care; without this foundation, true treatment effects are obscured by variable mortality attributable to poor baseline management. Improvements in supportive care have driven much of the observed mortality reduction in past outbreaks, often exceeding the contribution of any single investigational agent [49]. The uncontrolled use of multiple overlapping investigational therapies during prior outbreaks made it difficult to isolate drug-specific effects, underscoring the need for well-designed randomized trials planned before outbreaks occur. Vaccine strategies, such as ring vaccination and randomized timing or product comparisons, have demonstrated that it is possible to both limit spread and ethically evaluate efficacy simultaneously [50]. Research conducted during active HCID events faces distinct challenges, including difficulty obtaining informed consent, ethical tensions between care and research, regulatory delays, staff safety considerations, and limited capacity to analyze specimens in real time [51, 52]. NYC H + H/Bellevue has conducted simulation drills rehearsing consent processes and the operational deployment of investigational therapeutics, illustrating the value of maintaining research readiness between outbreaks.

### Staffing, training, and operational readiness

Effective BCU care depends as much on people and systems as it does on equipment and technology. The Ebola outbreak crystallized the necessity of critical care leadership within BCU teams. Before 2014, some centers, including Emory University Hospital, relied primarily on infectious diseases (ID) specialists as the managing service, with critical care support deployed on an ad hoc basis. The Ebola response prompted Emory to transition to an integrated ID-critical care staffing model, a shift that improved clinical decision-making, workflow efficiency, and overall care delivery [53, 54]. This transition reflected a broader recognition that HCID management in BCUs is fundamentally an intensive care activity requiring physician leadership with the operational expertise to coordinate multidisciplinary workflows during complex, high-risk interventions.

Intensive care nurses are the cornerstone of BCU operations, delivering continuous bedside care and helping lead the development and maintenance of a strong safety culture. Their frontline role includes implementing and teaching PPE protocols, evaluating their feasibility in practice, and providing feedback that informs procedures for infection prevention and control [55]. Hospital epidemiologists and infection prevention specialists oversee containment practices and real-time risk assessment, while respiratory therapists ensure adherence to infection control principles during airway management. Pharmacists play a critical operational role through just-in-time medication provisioning, minimizing waste and expiration by stocking automated dispensing cabinets in advance of patient arrival. Ethics consultants, social workers, and case management teams often participate virtually, enabling comprehensive, patient-centered care while limiting unnecessary exposure [56, 57]. 

During the 2014–2016 Ebola epidemic, institutions debated whether BCU staffing should be mandatory or volunteer-based. Most centers ultimately favored volunteer models, which promote psychological readiness and ensure that participating clinicians are genuinely committed to the unique demands of high-risk care, even if mandatory staffing offers stronger guarantees of workforce availability [58]. Several centers, including Johns Hopkins Hospital, refined their activation processes using rapid staffing protocols and controlled electronic sign-up systems to streamline deployment [6]. BCUs have consistently required higher nurse-to-patient ratios than conventional ICUs—as high as 4:1 during Ebola care—due to the time-intensive nature of donning and doffing protocols and the complexity of routine tasks such as medication administration, hygiene, and documentation under full PPE [59]. The COVID-19 pandemic reinforced that surge capacity planning must account not only for bed availability but also for sustainable nursing workloads, particularly when advanced therapies such as mechanical ventilation or ECMO are involved. As nurses are pulled from their regular ICU floors to staff the BCU, trickle-down effects are felt across the hospital, requiring careful and proactive backfill planning.

Training and preparedness are foundational to safe and effective BCU operations. Successful models incorporate recurring refresher training and high-fidelity simulation, supported by digital tools such as smartphone-accessible instructional videos, QR code-linked protocols, and shared NETEC resource repositories, alongside close integration with emergency department and infection prevention teams [60]. At RESPTCs, new staff must complete multiple onboarding sessions and demonstrate PPE competency before they can work in the BCU, and all staff thereafter attend quarterly training to maintain their permissions. These approaches promote a culture of readiness, allowing BCU teams to rapidly transition from routine operations to high-risk care while maintaining safety, competence, and mutual trust.

### Patient transport and systems integration

A major logistical challenge in HCID management is the safe transport of infected patients to BCUs capable of delivering definitive care. Several purpose-built transport systems have been developed to address this need. The U.S. Air Force’s Transport Isolation System (TIS) was developed before the 2014 Ebola outbreak and was strengthened during it, passing “Safe to Fly” testing in December 2014 and being formally accepted by U.S. Transportation Command (USTRANSCOM) in January 2015 [61]. Configurable to fly on multiple aircraft models including the C-130 H, C-130 J, and C-17, the TIS provides an isolated, medically capable environment for patient transport. The Department of State’s Portable Biocontainment Care Module (PBCM) similarly allows HCID patients to be transported aboard aircraft [62], and is related to the Aeromedical Biological Containment System (ABCS), developed from 2008 onward by the CDC and DoD in collaboration with Phoenix Air [63]. The 40-foot Negatively Pressurized CONEX (NPC) was later developed to meet the need for high-capacity airlift during the COVID-19 pandemic, with smaller NPC-Lite models configured for smaller aircraft.

While prior transport capabilities were primarily oriented toward international patient movement, the U.S. Department of Health and Human Services introduced its first domestic system for isolating and transporting HCID patients to RESPTCs in May 2025 [64]. This Portable Biocontainment Unit (PBCU) can be transported by air on a C-130 or L-100 aircraft to any U.S. airport, or by ground on a specialized trailer, allowing patients to remain isolated and receive continuous care in transit until arrival at a definitive care site. The development of this domestic capability represents an important milestone in closing the gap between outbreak detection and BCU-level care.

The emergence of mobile biocontainment platforms has broader implications for global health preparedness. In regions where constructing and sustaining fixed biocontainment hospitals is impractical, mobile units offer a scalable and cost-effective alternative that decentralizes containment while preserving clinical capability. However, the number of aircraft equipped for HCID transport remains limited, and the decontamination requirements between uses further constrain capacity. Expanding the transport network—both in terms of equipped aircraft and trained EMS agencies capable of managing HCID patients on the ground—remains a priority area for national and global preparedness systems.

### Military contributions to BCU care

Beyond originating the concept of BCUs at USAMRIID and developing HCID transport capabilities like the TIS and NPC, the U.S. military has focused longitudinal effort on improving detection, prophylaxis, treatment, and facilities for diseases that may threaten national security. For example, the DoD maintains the Global Emerging Infections Surveillance laboratory network to mitigate infectious disease threats [65] and it is also developing far-forward, point-of-care capabilities to improve disease detection beyond the lab setting [66]. On the vaccine and treatment front, the military has invested tremendous resources into creating new solutions for addressing HCIDs like hemorrhagic fevers [67, 68]. 

Outside of the research and development space, the military’s infrastructure for testing, just-in-time training, and constructing deployed hospital facilities were utilized during the 2013–2016 Ebola outbreak [69]. More recently, the U.S. military coordinated plans for staffing and transportation, set PPE requirements, selected medical countermeasures, and provided direct medical care during evacuation for patients affected by the 2026 Hantavirus outbreak on the MV Hondius ship [70]. Finally, the military’s rapidly deployable population of ready service-members means that it can bolster logistics and staffing anywhere, from domestic support to civil authorities in the homeland, as seen during the COVID-19 pandemic, to global health engagement operations overseas [71]. While these examples focus on BCU work from the U.S. military, militaries across the globe are actively involved in HCID research, preparedness, and response for perceived domestic, regional, and global disease threats [72, 73]. All of this work directly and indirectly improves effectiveness of military and civilian BCUs.

### Capability synergy, access, and future progress

**Fig. 2:**
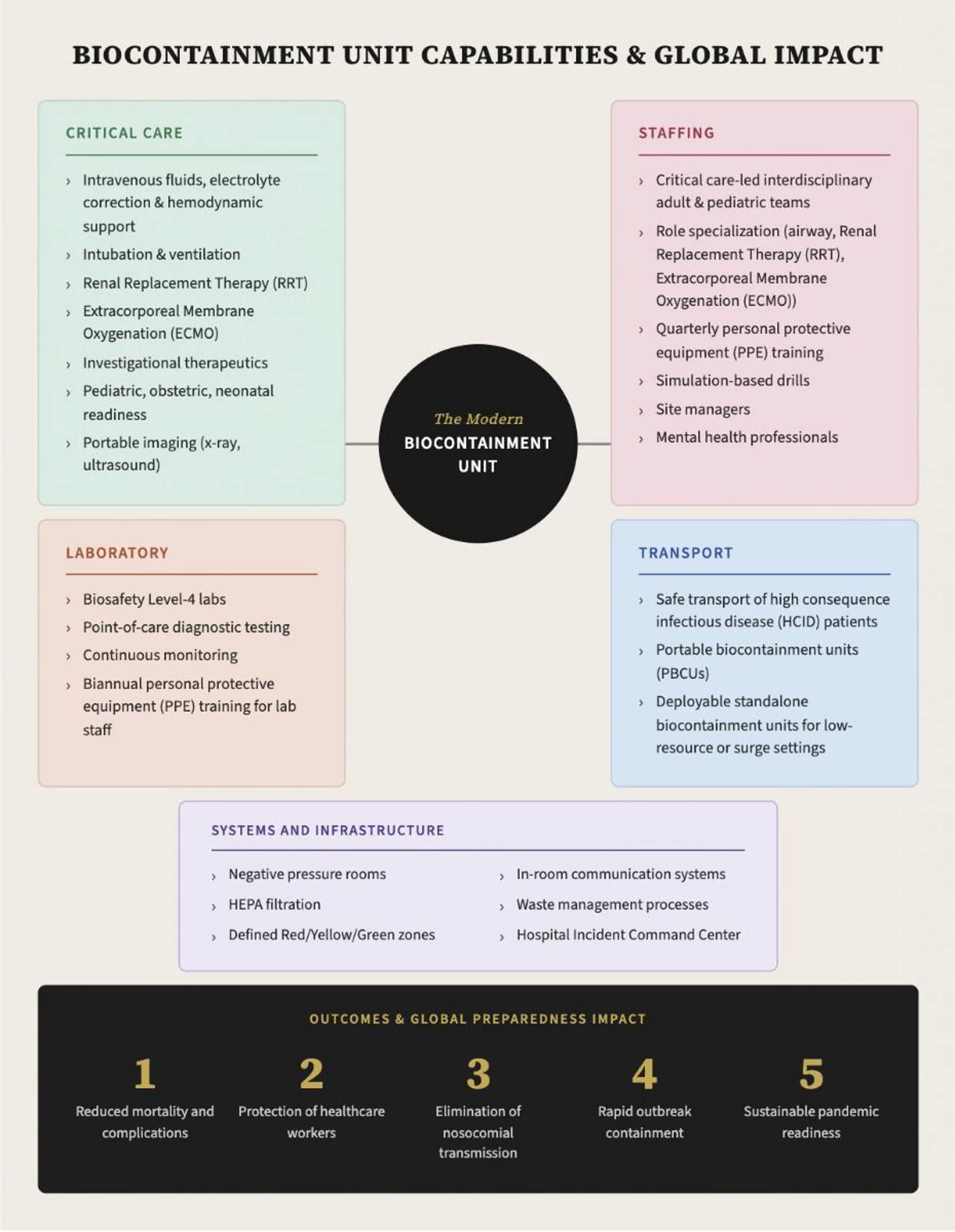
BCU Capabilities and Global Impact. The four capability domains of modern biocontainment units and their outcomes and global preparedness impact. BCU = Bio Containment Unit; IV=Intravenous; RRT= Renal Replacement Therapy; ECMO=Extracorporeal Membrane Oxygenation; PPE= Personal Protective Equipment; BSL=Biological Safety Level

The four core capability domains of modern BCUs—critical care, laboratory, staffing, and transport—are summarized in Fig. 2. Together these domains generate measurable outcomes: reduced mortality and complications, protection of healthcare workers, elimination of nosocomial transmission, rapid outbreak containment, and sustainable pandemic readiness. No single domain operates in isolation; the strength of a BCU’s response depends on how well these pillars are integrated and mutually reinforcing.

Equitable access to BCU-level care across patient populations also requires attention. While most units can accommodate both adults and children, some remain adult-only, pediatric-only BCUs are exceedingly rare, and not all units are designed to support bariatric patient care [25]. Addressing these gaps will require cross-training initiatives, shared staffing models, tele-critical care partnerships, and simulation-based preparedness focused on underrepresented patient groups. Similarly, persistent challenges in PPE design—particularly extreme heat burden on healthcare workers in high-temperature settings—continue to limit effective BCU care. Research into infrared-transparent fabrics, moisture-directing Janus materials, phase-change textiles, thermoelectric cooling systems, and graphene-based heat-dissipating materials aims to improve worker endurance and safety [74]. Alternative strategies such as patient isolation pods with built-in half-suits have been deployed to reduce PPE consumption, though these may impede certain clinical interventions [75]. Future progress in BCU care will require innovations that treat healthcare worker endurance and safety not as constraints to manage, but as central determinants of care quality.

## Conclusions

BCUs have evolved from isolation spaces into advanced units capable of delivering comprehensive critical care for patients with HCIDs. Experience from EVD, MVD, and COVID-19 outbreaks has demonstrated that strict infection control can coexist with high-quality, lifesaving care while protecting healthcare workers. However, we have seen that while these capabilities are within reach, many low-to-middle-income countries continue to experience difficulties implementing them. Maintaining this readiness requires sustained institutional commitment. Preserving lessons learned between outbreaks is essential. As emerging pathogens continue to challenge global health systems, BCUs will remain central to pandemic preparedness and the safe, effective delivery of critical care.

## Data Availability

No datasets were generated or analysed during the current study.

## Abbreviations

ABCS: Aeromedical biological containment system
ALIMA: The alliance for international medical action
BiPAP: Bilevel positive airway pressure
BCU: Biocontainment unit
BSL: Biosafety level
CDC: Center for disease control and prevention
CHMP: Committee for medicinal products for human use
COVID-19: Coronavirus disease 2019
CRRT: Continuous renal replacement therapy
DoD: Department of defense
DRC: Democratic republic of the congo
ECMO: Extracorporeal membrane oxygenation
ELSO: Extracorporeal life support organization
EMS: Emergency medical services
EVD: Ebola virus disease
FDA: U.S. food & drug administration
HCID: High consequence infectious disease
HEPA: High-efficiency particulate air
HHS: Health and human services
HIV: Human immunodeficiency virus
ICU: Intensive care unit
ID: Infectious diseases
JIHS: Japan institute for health security
KCDC: Korea centers for disease control and prevention
MCM: Medical counter measure
MERS: Middle east respiratory syndrome
MVD: Marburg virus disease
NCGM: National center for global health and medicine
NETEC: National emerging special pathogens training center
NGO: Non-governmental organization
NIID: National institute of infectious diseases
NPC: Negatively pressurized CONEX
NYC H + H/Bellevue: New York City Health and Hospitals / Bellevue
PARTNERS: Platform adaptive randomized trial for new and repurposed filovirus treatments
PBCM: Portable biocontainment care module
POCUS: Point of care ultrasound
PMH: Princess margaret hospital
PPE: Personal protective equipment
PREVAC: Partnership for research on ebola vaccines
RESPTCs: Regional Emerging special pathogen treatment centers
RRT: Renal replacement therapy
SARS-CoV-2: Severe acute respiratory syndrome coronavirus 2
STAKOB: The German permanent working group of competence and treatment centers for high consequence infectious diseases
TIS: U.S. Air force’s transport isolation system
UNICEF: United nations international children’s emergency fund
USAMRIID: U.S. Army’s medical research institute of infectious diseases
USTRANSCOM: United States transportation command
VA-ECMO: Veno-arterial extracorporeal membrane oxygenation
VHF: Viral hemorrhagic fever
